# Supplementary material for: Prevalence of Antibiotic-Resistant Seafood-Borne Pathogens in Retail Seafood Sold in Malaysia: A Systematic Review and Meta-Analysis
Source: Antibiotics (Basel). 2023 Apr 28;12(5):829. doi: 10.3390/antibiotics12050829 (PMC10215680; doi:10.3390/antibiotics12050829)
Supplement: Supplementary file 1 [file antibiotics-12-00829-s001.zip › antibiotics-2300404-supplementary.pdf]

Aeromonas only = 57 (study 2)

Antimicrobials	Article 2		
Ampicillin	57	57	100.00
Bacitracin	47	57	82.46
Carbenicillin	55	57	96.49
Ceftriaxone	9	57	15.79
Cephalothin	22	57	38.60
Cefoperazone	8	57	14.04
Chloramphenicol	3	57	5.26
Erythromycin	54	57	94.74
Gentamicin	29	57	50.88
Kanamycin	26	57	45.61
Nalidixic acid	14	57	1
Nofloxacin	6	57	2
Streptomycin	46	57	3
Tetracycline	24	57	4
Chloramphenicol	4	11	5
Clindamycin	11	11	6
Penincillin G	4	11	
Rifampicin	11	11	
Spectinomycin	3	11	
Tetracycline	8	11	
Amikacin	121	235	
Amoxicilin-clavulanic acid	3	235	
Ampicillin	200	235	
Ampicillin-sulbactam	36	235	
Cefotaxime	87	235	
Ceftazidime	28	235	
Cephalothin	21	235	
Chloramphenicol	10	235	
Ciprofloxacin	3	235	
Doxycycline	2	235	
Gentamicin	9	235	
Imipenem	19	235	
Kanamycin	82	235	1
Levofloxacin	2	235	2
Nalidixic acid	14	235	3
Penincillin G	91	235	4
Streptomycin	36	235	5
Tetracycline	19	235	6
Trimethoprim sulfamethoxazole	8	235	7
			8
			9

10
11
12
13
14
15
16
17
18
19

Antimicrobials	Article 4	Article 7		Article 8	Article 9
Chloramphenicol	4		4	15	106
Clindamycin	7	4	11	3	
Penincillin G		4	4	55	145
Rifampicin	7	4	11		36
Spectinomycin	3		3		
Tetracycline	4	4	8		
				1	86
					28
**Salmonella = 11 (studies 4 and 7)**					
Antimicrobials				21	
Chloramphenicol	4	11	36.36364		
Clindamycin	11	11	100	6	4
Penincillin G	4	11	36.36364	3	
Rifampicin	11	11	100		
Spectinomycin	3	11	27.27273	2	
Tetracycline	8	11	72.72727		
				7	2
					19
Clindamycin	100				82
Rifampicin	100			1	1
Spectinomycin	27				14
				62	29
				36	
				6	13
Trimethoprim sulfamethoxazole					8

vibrio para = 235 (studies 8 and 9)

Antimicrobials	Article 8	Article 9			
Amikacin	15	106	121	235	51.49
Amoxicilin-clavulanic acid	3		3	235	1.28
Ampicillin	55	145	200	235	85.11
Ampicillin-sulbactam		36	36	235	15.32
Cefotaxime	1	86	87	235	37.02
Ceftazidime		28	28	235	11.91
Cephalothin	21		21	235	8.94
Chloramphenicol	6	4	10	235	4.26
Ciprofloxacin	3		3	235	1.28

Doxycycline	2		2	235	0.85
Gentamicin	7	2	9	235	3.83
Imipenem		19	19	235	8.09
Kanamycin		82	82	235	34.89
Levofloxacin	1	1	2	235	0.85
Nalidixic acid		14	14	235	5.96
Penincillin G	62	29	91	235	38.72
Streptomycin	36		36	235	15.32
Tetracycline	6	13	19	235	8.09
Trimethoprim sulfamethoxazole		8	8	235	3.40

	Vibrio = 378		
1	Amikacin	208	378
2	Amoxicilin-clavulanic acid	1	378
3	Ampicillin	335	378
4	Ampicillin-sulbactam	72	378
5	Carbenicillin	11	378
6	Cefepime	4	378
7	Cefotaxime	168	378
8	Cefoxitin	15	378
9	Ceftazidime	112	378
10	Cefuroxime	62	378
11	Cephalothin	65	378
12	Cefazolin	101	378
13	Chloramphenicol	48	378
14	Ciprofloxacin	16	378
15	Erythromycin	22	378
16	Gentamicin	17	378
17	Imipenem	11	378
18	Kanamycin	149	378
19	Levofloxacin	15	378
20	Nalidixic acid	11	378
21	Ofloxacin	3	378
22	Oxytetracycline	39	378
23	Penincillin G	151	378
24	Piperacillin	43	378
25	Piperacillintazobactam	19	378
26	Streptomycin	32	378
27	Tetracycline	59	378
28	Trimethoprim sulfamethoxazole	30	378
29	Teicoplanin	11	378

14 antibiotics

Antimicrobials	Article 6		
Amikacin	95	185	51.4
Ampicillin	151	185	81.6
Ampicillin-sulbactam	3	185	1.6
Cefotaxime	69	185	37.3
Ceftazidime	28	185	15.1
Chloramphenicol	8	185	4.3
Gentamicin	21	185	11.4
Imipenem	4	185	2.2
Kanamycin	52	185	28.1
Levofloxacin	17	185	9.2
Nalidixic acid	35	185	18.9
Oxytetracycline	35	185	18.9
Tetracycline	31	185	16.8
Trimethoprim sulfam	7	185	3.8

**Red prawn**				**Banana pra**
	97			88
	37	97	38.14	68
	77	97	79.38	74
	0	97	0.00	3
	25	97	25.77	44
	1	97	1.03	27
	5	97	5.15	3
	1	97	1.03	20
	1	97	1.03	3
	8	97	8.25	44
	0	97	0.00	17
	0	97	0.00	35
	10	97	10.31	25
	0	97	0.00	7
	6	97	6.19	25

Antimicrobials	Article 3
Chloramphenicol	4
Streptomycin	10
Sulfamethoxazole	10
Tetracycline	2

awn

88	77.27
88	84.09
88	3.41
88	50.00
88	30.68
88	3.41
88	22.73
88	3.41
88	50.00
88	19.32
88	39.77
88	28.41
88	7.95
88	28.41

**Antibiotic**	**Positive**
Amikacin (30ug)	13
Ampicillin (10ug)	22
Cefazolin (30ug)	22
Cefotaxime (30ug)	1
Cefoxitin (30ug)	2
Ceftazidime (30ug)	1
Cefuroxime sodium (pare	15
Cephalothin (30ug)	16
Ciprofloxacin (5ug)	3
Gentamicin (10ug)	2
Penicillin G (10 unit)	26
Piperacillin (100ug)	12
Piperacillintazobactam (1	5

Total isolates
28
28
28
28
28
28
28
28
28
28
28
28
28
